# Status of Mitochondrial Oxidative Phosphorylation during the Development of Heart Failure

**DOI:** 10.3390/antiox12111941

**Published:** 2023-10-31

**Authors:** Sukhwinder K. Bhullar, Naranjan S. Dhalla

**Affiliations:** Institute of Cardiovascular Sciences, St. Boniface Hospital Albrechtsen Research Centre, Department of Physiology and Pathophysiology, Max Rady College of Medicine, University of Manitoba, Winnipeg, MB R2H 2A6, Canada; sbhullar@sbrc.ca

**Keywords:** oxidative phosphorylation (OXPHOS), mitochondrial Ca^2+^-overload, mitochondrial electron transport chain, heart failure, cardiac dysfunction

## Abstract

Mitochondria are specialized organelles, which serve as the “Power House” to generate energy for maintaining heart function. These organelles contain various enzymes for the oxidation of different substrates as well as the electron transport chain in the form of Complexes I to V for producing ATP through the process of oxidative phosphorylation (OXPHOS). Several studies have shown depressed OXPHOS activity due to defects in one or more components of the substrate oxidation and electron transport systems which leads to the depletion of myocardial high-energy phosphates (both creatine phosphate and ATP). Such changes in the mitochondria appear to be due to the development of oxidative stress, inflammation, and Ca^2+^-handling abnormalities in the failing heart. Although some investigations have failed to detect any changes in the OXPHOS activity in the failing heart, such results appear to be due to a loss of Ca^2+^ during the mitochondrial isolation procedure. There is ample evidence to suggest that mitochondrial Ca^2+^-overload occurs, which is associated with impaired mitochondrial OXPHOS activity in the failing heart. The depression in mitochondrial OXPHOS activity may also be due to the increased level of reactive oxygen species, which are formed as a consequence of defects in the electron transport complexes in the failing heart. Various metabolic interventions which promote the generation of ATP have been reported to be beneficial for the therapy of heart failure. Accordingly, it is suggested that depression in mitochondrial OXPHOS activity plays an important role in the development of heart failure.

## 1. Introduction

The heart is the most active organ in the body and requires a significant amount of energy in the form of adenosine triphosphate (ATP) to sustain its vital function continuously. Despite having a limited ATP storage capacity, the heart has a remarkably efficient and reliable energy production system facilitated by the abundant presence of mitochondria in cardiomyocytes. Mitochondria, known as the “powerhouse” in the heart, perform a crucial role in generating ATP through the process of oxidative phosphorylation (OXPHOS) [1,2,3,4,5]. Furthermore, these organelles are involved in the exchange and synthesis of metabolites, calcium storage, the production of reactive oxygen species (ROS), as well as cell survival and death signals, all of which are critical for regulating cardiac function in health and disease. Additionally, mitochondria have been shown to participate in various signaling pathways associated with the regulation of oxidative stress, inflammation, mitophagy, calcium handling, and apoptosis, which are fundamental to inducing cardiac dysfunction under diverse pathophysiological conditions [6,7,8,9,10,11]. Since mitochondria generate ATP upon the oxidation of different substrates, their function is dependent upon the type of substrate availability as well as the status of glycolysis, lipolysis and proteolysis in cardiomyocytes [12,13,14,15]. In particular, these organelles regulate metabolic pathways such as the tricarboxylic acid cycle and the beta-oxidation of free fatty acids, which contribute to the electron transport chain in the OXPHOS complex and impact processes like ROS production, redox state, and apoptosis [16,17,18,19].

Mitochondrial abnormalities associated with cardiac dysfunction include disruption in the process of energy generation, cation accumulation, ROS production, aldehydic load, and intracellular signaling. Since mitochondria play a fundamental role in sustaining cardiac contractility, the impairment of their function has been implicated in several cardiovascular diseases, in which a decrease in ATP production has been shown to cause a depletion of cardiac energy stores [19,20,21,22,23,24,25,26,27,28,29]. Risk factors of cardiovascular diseases, such as ischemia/reperfusion injury, hypertension, ventricular hypertrophy, cardiomyopathies, atherosclerosis, metabolic syndrome, and diabetic hyperglycemia, have been reported to produce mitochondrial dysfunction [30,31,32,33,34,35,36,37]. The role of mitochondria in producing ROS is critical because they function as redox messengers when generated within normal levels, but excessive ROS production can lead to oxidative stress and ultimately result in cell death. It has been indicated that oxidative stress, in addition to producing mitochondrial dysfunction, can cause the increased production of pro-inflammatory cytokines and the activation of fibroblasts in the extracellular matrix. These alterations result in interstitial fibrosis and passive stiffness of the myocardium. However, extensive work is required to establish the exact cause–effect relationship among myocardial oxidative stress, myocardial inflammation and mitochondrial dysfunction during the development of heart failure. Nonetheless, mitochondrial oxidative stress has been shown to increase Ca^2+^-influx, which worsens cardiomyocyte relaxation and elevates the left ventricle filling pressure, along with proteolytic damage to the heart [38,39,40,41,42]. The intrinsic compensatory mechanisms such as the level of intracellular Ca^2+^ and antioxidant system that typically control the oxidation of substrates and energy production in mitochondria also fail to offset the depletion of myocardial energy stores during the development of cardiac dysfunction [43,44,45,46]. 

Considering the high energy demand of the cardiac excitation–contraction and relaxation cycle, patients with abnormalities in the mitochondrial OXPHOS are at a higher risk of developing heart disease [34,35,44,47]. Different defects within the mitochondrial OXPHOS system, which can arise due to genetic or environmental factors, have been implicated in various cardiac disorders, including ischemia/reperfusion injury, hypertension, arrhythmias, cardiac hypertrophy, cardiomyopathies, and heart failure [3,30,35,36]. Thus, impaired OXPHOS plays a significant role in the onset of various cardiovascular diseases, which can manifest differently depending on the underlying cause and stage of heart disease [9,48,49,50]. Although abnormalities in mitochondrial OXPHOS have long been observed in cases of heart failure and other cardiac pathologies, the underlying causes of these abnormalities remain poorly understood. It is, therefore, the objective of this article to provide a comprehensive overview of the impact of impaired mitochondrial OXPHOS during the development and progression of heart failure as a consequence of various pathological conditions. Several components of the mitochondrial OXPHOS system as well as their functions in the heart will be described. In addition, some pharmacological and metabolic interventions aimed at OXPHOS pathway defects as targets, which prevent or treat heart failure and enhance patient survival, are also summarized. 

## 2. Function of Mitochondrial OXPHOS in the Heart

Mitochondria are specialized organelles that feature two membranes and their own DNA system. Their inner membrane, characterized by numerous infoldings (cristae), hosts several proteins, including enzymes that facilitate mitochondrial OXPHOS. The controlling of this metabolic process depends on a variety of factors, including intrinsic kinetic parameters and the regulation of various enzymes, the architecture network as well as intermediate substrate concentrations under steady-state conditions [51,52]. OXPHOS complexes [53,54], namely complex I (NADH/ubiquinone oxidoreductase), complex II (succinate ubiquinone oxidoreductase), complex III (ubiquinol cytochrome c oxidoreductase), complex IV (cytochrome c oxidase), and complex V (ATP synthase) are located on the inner membrane [55,56,57,58,59,60]. These complexes, collectively known as the electron transport chain, transfer electrons from donors generated by the tricarboxylic acid cycle or fatty acid oxidation [61] for the generation of ATP. The nuclear and mitochondrial genomes regulate these complexes, with mitochondrial genes playing a major role in assembling the core complexes within the mitochondria. The cardiac cells need a lot of ATP and have mitochondria occupying approximately 20–40% of their volume [62]. These organelles produce about 6 kg of ATP daily through OXPHOS-associated electron transport mechanisms in the human heart [5]. In a normal heart, fatty acid, glucose, and a ketone body enter cardiomyocytes and are transported into mitochondria in various forms [14,15,63]. These are catabolized in the mitochondria to acetyl coenzyme A (acetyl-CoA) for entering the tricarboxylic cycle and then undergo a series of redox reactions in the electron transport chain. This process is associated with the production of NADH and FADH2, which are then oxidized by NADH dehydrogenase and succinate dehydrogenase in the inner membrane. OXPHOS complex I and complex II receive these electrons from the donors, after which Coenzyme Q (CoQ) transports them to complex III [57,64,65]. Eventually, the electrons are transferred to the hydrophilic heme protein cytochrome C and then to complex IV [57]. The electron transport chain then creates a proton-motive force and, simultaneously, protons are pumped into the mitochondrial intermembrane space against a concentration gradient (ΔpHm) [49,66]. 

The movement of electrons in the respiratory chain creates a negative charge inside the mitochondrial matrix and is termed as mitochondrial membrane potential (ΔΨm) [67]. Protons then re-enter the mitochondrial matrix via complex V due to the proton gradient to generate ATP from ADP [57]. Phosphocreatine functions as an energy buffer that facilitates intracellular ATP transfer, whereas the clusters of mitochondrial electron transport complexes combine to form supercomplexes, which are crucial in regulating electron flow within mitochondria [68,69,70]. It is noteworthy that the inner mitochondrial membrane is mostly impenetrable to cations and small molecules, thus making proton pumping a critical step in this conversion process [71]. During the OXPHOS process in mitochondria, superoxide anions are produced by about 2% of electrons passing through the electron transport chain in complexes I, II, and III [72,73], but these are rapidly altered or dismutated by superoxide dismutase (MnSOD and CuZnSOD) to form hydrogen peroxide, which is then broken down into water by antioxidant enzymes, including catalase, glutathione peroxidase and peroxiredoxins [74,75,76]. The ROS signaling molecules are the byproducts of oxygen metabolism and affect oxygen-sensing mechanisms like gene expression. It is also pointed out that cardiolipin is a critical phospholipid which is pivotal in stabilizing mitochondrial OXPHOS complexes and facilitating supercomplexes’ formation within the electron transport chain and thus precisely regulating ATP production in the mitochondria. The OXPHOS system complexes I-V and molecules like CoQ and cardiolipin work together to ensure the optimal functioning of the mitochondrial energy-producing system [77,78,79,80]. However, it is pointed out that in a study concerning the role of mitochondrial supercomplexes in maintaining OXPHOS activity, Milenkovic and coworkers [81] failed to demonstrate any change in the mitochondrial bioenergetic capacity under conditions associated with a major loss of respirasomes.

## 3. Impact of Impaired OXPHOS in the Pathogenesis of Heart Failure

### 3.1. Bioenergetics and OXPHOS Capacity

Several studies on both human and animal heart disease models have revealed a notable decline in the cellular ATP and phosphocreatine content, indicating an altered energy metabolism in the heart. As a result, different abnormalities observed in heart disease, including altered nutrient usage, reduced OXPHOS activity, increased oxidative stress, and aberrant calcium handling and mitochondrial dynamics, lead to alterations in energy metabolism, which ultimately result in the irreversible deterioration of heart function [82,83,84,85,86,87,88,89,90]. An imbalance in the ATP supply triggered by pathological stimuli under conditions such as left ventricular remodeling, chamber dilation, and hypertrophy can worsen the progression of heart disease. Such defects may increase the energy demand while reducing the energy supply, leading to altered bioenergetics in the diseased heart. These changes impair OXPHOS and associated activities, including the creatine kinase energy-transfer mechanism, elevating free adenosine diphosphate (ADP) levels, and decreasing the ATP content during the later stages of heart disease [44,45,91,92]. Various studies have referred to heart failure as an energy-deprived state characterized by a decline in ATP production and driven mainly by impaired OXPHOS. The appropriate functioning of the heart relies heavily on the efficient mitochondrial oxidative metabolism to maintain ATP production. Thus, any malfunction or disruption in the role of OXPHOS electron transport chain complexes for the production of energy in mitochondria may lead to an imbalance in cardiac cell metabolism.

In the diseased heart, varying degrees of changes in substrate utilization, a reduction in the electron transport chain activity, depression in both ATP and phosphocreatine content, and attenuation in the rate of ATP transfer to phosphocreatine have been observed [93]. Mitochondrial dysfunction enhances ROS levels through electron transport chain-mediated ROS production due to the defective regeneration of NADPH and the excessive levels of ROS are known to cause detrimental effects in the myocardium. These ROS molecules are known to alter proteins, DNA, and lipids, leading to oxidative damage in the heart [37,72,94,95,96]. Additionally, an overload of ROS can result in the abnormal opening of the mPTP, releasing detrimental substances and leading to the swelling of the mitochondria, ruptured membranes, and triggering inflammation, apoptosis, and cell damage [97,98]. Furthermore, an overabundance of ROS can deplete the intracellular redox pool, impair cellular Ca^2+^ handling, cation channel activities, and ROS-mediated redox signaling pathways [99,100,101]. Although mitochondrial ROS signaling is important for the regulation of the oxidative metabolism, muscle contraction, and calcium transport [102,103,104,105], an imbalance between the production of ROS and the endogenous antioxidant system has been shown to produce oxidative stress. In fact, mitochondrial dysfunction increases oxidative stress through alterations in the tricarboxylic acid cycle and ATP synthase as well as cardiolipin degradation and mitochondrial electron leakage, which thus is considered to cause significant damage to the myocardium depending on the type and stage of heart disease [69]. Furthermore, heightened levels of mitochondrial oxidative stress markers have been reported to produce increased amount of oxidants in the electron transport chain at complex I in the diseased heart [106,107].

The extent of mitochondrial damage has been suggested as a key factor when determining myocardial injury due to myocardial infarction during progression to heart failure [36]. It is pointed out that acute changes such as cardiogenic shock and ischemia-reperfusion injury in myocardial infarction should be differentiated from chronic alterations associated with pathological hypertrophy and cardiac remodeling. Ischemia-reperfusion injury, as a consequence of coronary heart disease, dramatically increases mitochondrial permeability leading to the dissipation of electron and proton gradients, the dysregulation of mitochondrial calcium homeostasis, and the release of superoxide radicals which lead to myocardial cell death [108]. Furthermore, mitochondrial supercomplexes lose their integrity as the electron transport chain subunits degrade due to ischemia/reperfusion injury, leading to the impairment of mitochondrial function [109]. It was observed that a decrease in the complex I subunit and an increase in the complex II subunits occur, suggesting a redirection of the electron input through complex II [110]. During the reperfusion phase of the ischemia-reperfusion injury, an abrupt elevation in ROS levels can induce myocardial cell damage resulting in cellular death through necrosis [111]. The overproduction of ROS triggered by ischemia as well as ischemia-reperfusion has also been considered to initiate apoptosis in cardiac cells, which is a significant contributing factor for the development of heart failure [112]. It is noteworthy that mitochondrial respiration is a crucial determinant of the functional status of the OXPHOS system [113,114,115]. A study on dogs with chronic heart failure induced by intracoronary embolization revealed that the mitochondrial state-3 respiration and mitochondrial membrane potential in the failing heart were lower than those in the healthy heart. This decline in oxygen consumption by mitochondria and reductions in the membrane potential were associated with alterations in the OXPHOS complexes. In fact, dysfunction in the mitochondrial tricarboxylic acid cycle was observed to be closely linked to heart failure [116,117]. Another study involving myocardial infarction in mice demonstrated that chronic heart failure causes a decrease in the mitochondrial OXPHOS capacity due to decreased levels of succinyl-CoA in the myocardium. Furthermore, administering 5-aminolevulinic acid to infarcted mice was found to restore the succinyl-CoA levels and OXPHOS capacity by inducing excessive heme synthesis, potentially attenuating the progression of heart failure [118]. A recent study with cardiac tissue samples from heart failure patients has also revealed a dysfunction in succinyl-CoA metabolism [119].

### 3.2. Genetic Regulation of OXPHOS

It is pointed out that the DNA damage response and RNA polymerase II pausing pathway are significantly downregulated in failing human hearts, as well as primate and murine hearts, following myocardial infarction [120]. In a mouse model, the cardiac-specific inactivation of LARP7 (La ribonucleoprotein domain family member 7) resulted in decreased oxidative phosphorylation, mitochondrial biogenesis impairment and elevated oxidative stress, ultimately leading to heart failure. These irregularities, as well as the reduced deacetylase activity of SIRT1 (silent mating-type information regulation 2 homolog 1), which is responsible for the transcription of genes related to the mitochondrial OXPHOS system and energy metabolism, were shown to reduce cardiac function. The restoration of LARP7 expression in the infarcted heart through adenovirus-mediated LARP7 expression or by a small molecule ATM inhibitor has been shown to improve the function of the injured heart [65,121]. Decreased PGC1 (Peroxisomal proliferator-activated receptor gamma coactivator 1)-α levels and reduced nuclear genome-encoded OXPHOS complexes have been observed in animal models of heart failure [122,123]. Since heart failure with a preserved ejection fraction (HFpEF) and heart failure with a reduced ejection fraction (HFrEF) are metabolically distinct, changes in the OXPHOS gene transcripts have been shown to characterize these differences. The transcriptome analysis of ventricular tissue from patients with HFpEF and HFrEF has revealed the involvement of elevated genes in OXPHOS [124] and impaired complex1-mediated mitochondrial respiration in permeabilized cardiac fibers in the HFpEF condition [125]. Furthermore, the degradation of cardiolipin due to oxidative stress, a significant increase in mitochondrial electron leakage, and reduced levels of CoQ have been observed in patients and animals with heart failure, indicating malfunction in the electron transport chain and oxidative phosphorylation’s ability to produce sufficient ATP in the failing heart [126,127]. A sufficient intake of linoleic acid in heart failure has been demonstrated to increase cardiolipin levels, improve mitochondrial OXPHOS activity, and enhance left ventricular function [128].

It may be noted that the OXPHOS complexes are encoded by both the nuclear and mitochondrial genomes as various studies on gene expression have revealed that individuals afflicted with heart disease possess reduced levels of mitochondrial metabolic genes and proteins. In this regard, mutations in genes regulating mitochondrial proteins were found to adversely impact energy production, decrease mitochondrial function, increase ROS production, and result in the development of cardiomyopathy [129]. Patients diagnosed with dilated cardiomyopathy also exhibit significant alterations in their metabolic pathways, specifically, the enzymes related to OXPHOS and the tricarboxylic acid cycle were down-regulated for complex III at both transcriptional and proteomic levels. Furthermore, the activities of complex III and IV in the left ventricular tissue from these patients were depressed [129,130,131]. It has also been shown that dilated hypertrophy influences energy generation by the mitochondria, which can alter the transcript levels of nuclear DNA- and mitochondrial DNA (mtDNA)-encoded mitochondrial genes and result in the reduced production of new mitochondria, impaired mitochondrial OXPHOS, and increased ROS production [132]. On the other hand, hypertrophic cardiomyopathy was found to be associated with cytochrome C deficiency leading to death [132,133]. Mitochondrial defects in the electron transport chain have also been implicated in the pathogenesis of diabetic cardiomyopathy [134]. Since the dilated hypertrophy, hypertrophic cardiomyopathy, and diabetic cardiomyopathy have different oxidative stress patterns [135,136], it is likely that differences in the profiles of mitochondrial abnormalities may be a consequence of differences in ROS production in these pathological conditions.

### 3.3. Structural Changes in Mitochondrial Network

Alterations in the mitochondrial ultrastructure and function, including reductions in the activities of respiratory chain enzymes (complexes I to IV) and capacity for OXPHOS, are commonly observed in patients with heart failure, although these may not manifest until the later stages of the disease. In this regard, chronic hypertrophy without systolic dysfunction has been shown to be associated with normal or improved mitochondrial function in both animals and humans [130,137,138,139,140,141]. On the other hand, impaired OXPHOS in adverse ventricular remodeling due to volume overload was observed before any signs of systolic dysfunction or decompensation were detected [142]. Some studies have shown that OXPHOS rates tend to increase during the early stages of cardiac hypertrophy, but decline as the condition progresses towards heart failure [26,143]. A diminished expression of OXPHOS components was reported to result in a decline in mitochondrial respiration in heart failure and cardiomyopathies [82,144]. These changes in mitochondrial function may be a consequence of oxidative stress, which in heart failure arises from various sources of ROS such as the activation of NADPH and monoamine oxidase as well as mitochondrial complexes I, II, and III, which are considered to play a major role in ROS production. Compared to healthy hearts, cardiomyocytes exhibit a significant increase in ROS levels within the mitochondrial matrix in failing hearts [116,117,145]. The interaction between a small amount of ROS and mitochondrial components leads to mitochondrial dysfunction, which produces more ROS, and further damages the mitochondria, impairs contractile dysfunction, and worsens heart failure [146,147]. A marked increase in oxidative stress in heart failure may also be a consequence of the depletion of different antioxidant enzymes and antioxidants in the failing heart [148].

Malfunctions in the respiratory chain can initiate oxidative stress and prompt the emergence of cardiac hypertrophy. The loss of mitochondrial ribosomal protein S5 (MRPS5/uS5m) in the developing heart leads to cardiac defects and embryonic lethality, while postnatal loss impairs mitochondrial protein translation and OXPHOS during the development of cardiac hypertrophy and heart failure [149]. Since the structural and functional changes in the damaged mitochondrial network are critical, both fusion and fission processes are required in distributing protein and DNA [150,151]. The disruption of these processes can lead to mitochondrial damage and cell death. It should be pointed out that fusion is necessary for maintaining OXPHOS and energy levels, protecting against oxidizing molecules, and preserving the mitochondrial integrity [152,153]. Fusion proteins, Mfn-1, Mfn-2, and OPA-1 are essential for preserving mitochondrial integrity, while their suppression can lead to dilated cardiomyopathy and contractile abnormalities [154], and increase apoptosis and the fragmentation of the mitochondria [155,156,157,158,159]. Their deletion in a mouse heart was observed to result in an abnormal mitochondrial morphology and mitochondrial fragmentation leading to ventricular wall thickening and an increase in cardiac mass, accompanied by eccentric hypertrophy [160]. On the other hand, excessive fission leads to a loss of mitochondrial mass, impaired OXPHOS and ATP deficits, permeabilization, cytochrome C release, and apoptosis [161]. The deficiency of the fission protein, dynamin-related protein 1 (Drp1), which is highly expressed in the heart, exhibited lethal dilated cardiomyopathy with ventricular wall thinning and a reduced ejection fraction [162].

## 4. Stage- and Type-Dependent Changes in OXPHOS in Heart Failure

### 4.1. General Considerations

Various studies have found that defects in the electron transport complexes and other components of the mitochondrial OXPHOS system may vary depending on the cause (type) and severity (stage) of heart failure. When OXPHOS is impaired, it can result in the irregular production of ROS, which in turn leads to inflammation and oxidative stress, contributing to a range of cardiac abnormalities, such as cardiac hypertrophy, arrhythmias, and cardiomyopathy during the development of heart failure [26,27,48,49,163]. Reduced mitochondrial respiratory rates and changes in the OXPHOS function may signal the beginning of heart failure. These alterations commonly involve complex I-linked respiration, fatty acid oxidation, and the OXPHOS system in human hearts [113,114]. Further, it has been revealed that lower ADP-dependent respiratory rates are observed in dilated cardiomyopathy, pressure overload, or myocardial infarction [82,83,164,165]. It is also pointed out that some investigators have failed to detect any changes in heart failure [141], whereas others have demonstrated a depression in the OXPHOS activity at early stages [114]. It appears that changes in mitochondrial OXPHOS activities are dependent upon the type and stage of heart failure.

### 4.2. Cardiomyopathic Hamster Heart Failure

**Figure 1:**
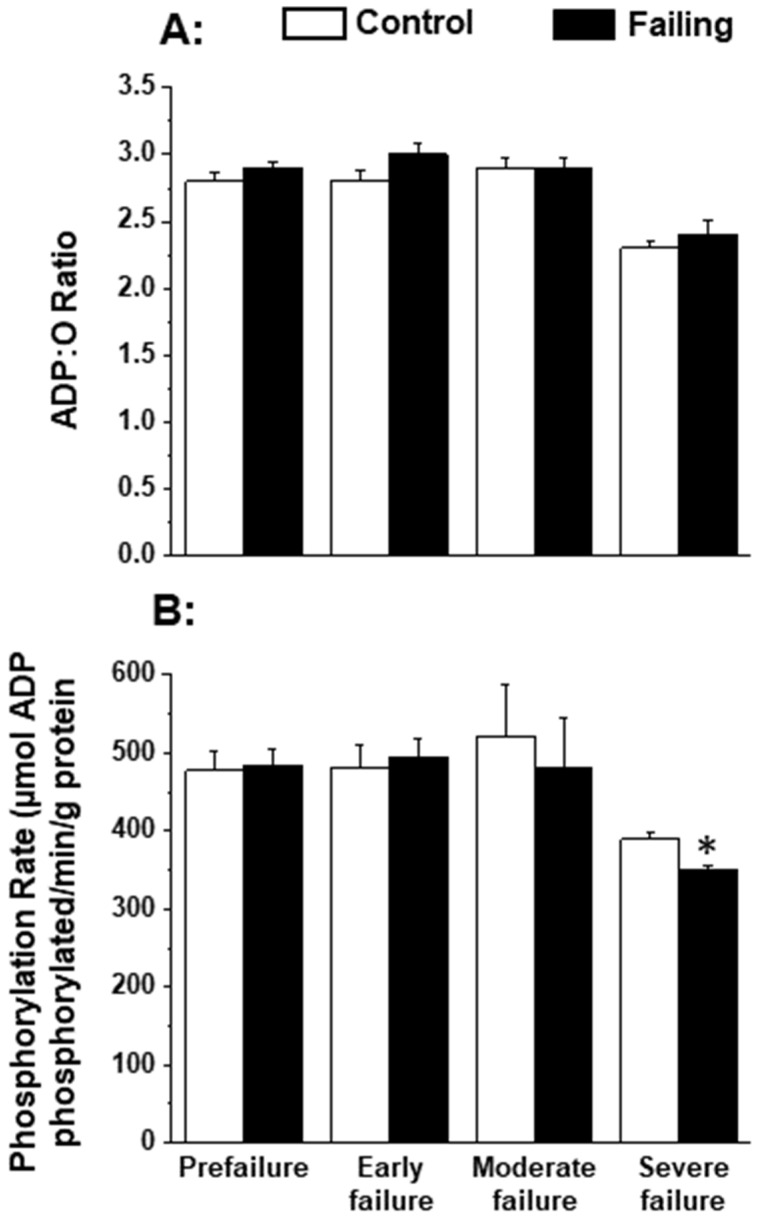
Oxidative phosphorylation rate (**B**) and ADP:O ratio (**A**) in by heart mitochondria in cardiomyopathic hamsters (UM-X7.1) at different stages of congestive heart failure. Data are based on the results in our articles [24,163]. The isolation of mitochondria was carried out using 10 mM ethylenediaminetetra-acetic acid (EDTA) [163]. Each value is mean of ± SE of four to six experiments. Mitochondria were isolated by pooling 4 hearts for each experiment. The substrate employed was 1.5 mM pyruvate plus 0.3 nM malate. Different groups of cardimyopathyic hamsters were selected on the basis of their age: Prefailure (90 to 100 days), Early failure (120 to 160 days), Moderate failure (160 to 200 days), and Severe failure (200 to 280 days). Age-matched control hamsters were used for each group. The depression in phosphorylation rate at severe stages of heart failure was due to a significant decrease in the state 3 respiratory rate without any changes in the state 4 respiration [163]. * *p* < 0.05. ADP, adenosine diphosphate.

**Figure 2:**
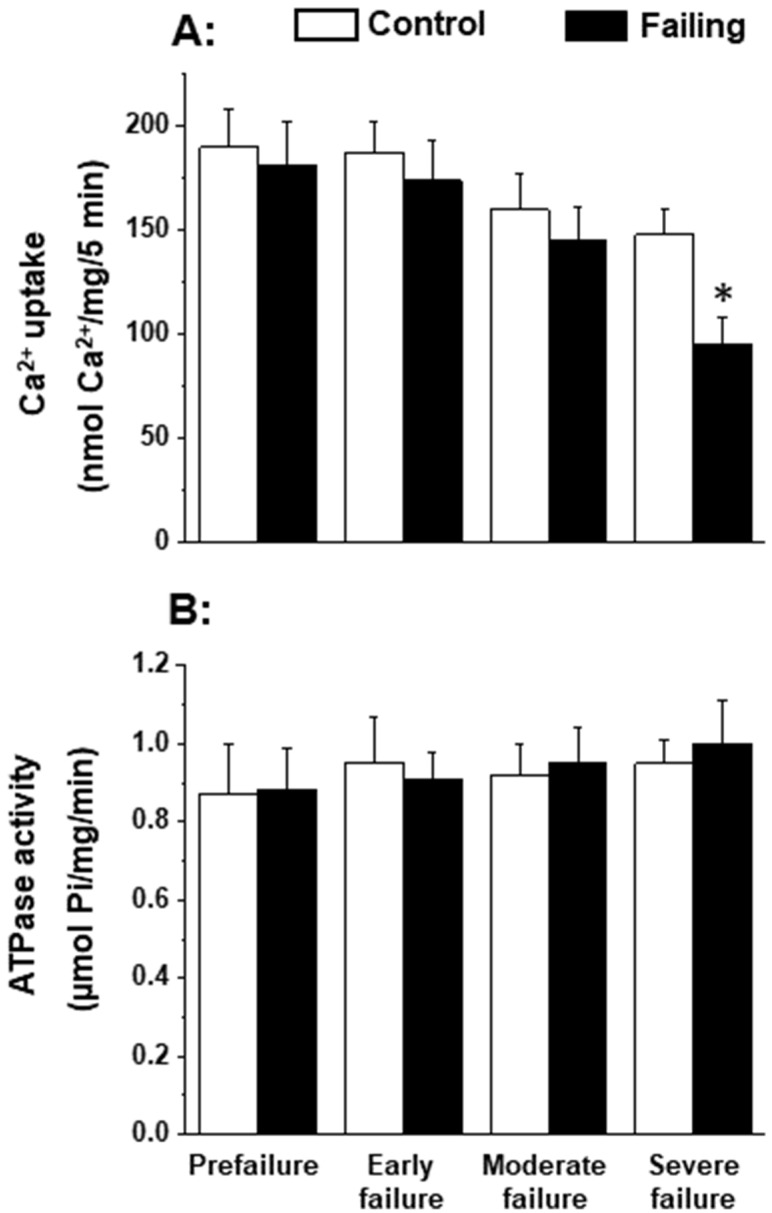
Mitochondrial Ca^2+^-uptake (**A**) and ATPase (**B**) activities in cardiomyopathic hamsters (UM-X7.1) at different stages of congestive heart failure. Data are based on the results in our articles [24,163]. It is pointed out that mitochondria were isolated using a medium containing 10 mM ethylenediaminetetra-acetic acid (EDTA) [163]. Each value is mean of ± SE of four to six experiments. Mitochondria were isolated by pooling 4 hearts for each experiment. Different groups of cardimyopathyic hamsters were selected on the basis of their age: Prefailure (90 to 100 days), Early failure (120 to 160 days), Moderate failure (160 to 200 days), and Severe failure (200 to 280 days). Age-matched control hamsters were used for each group. * *p* < 0.05.

Since the patterns of oxidative stress have been shown to be different with respect to acute heart failure and chronic heart failure [166,167], it is likely that alterations in mitochondrial OXPHOS may also be dependent upon the stage of heart failure. In order to gain some information in this regard, we employed cardiomyopathic hamsters (UM-X7.1) for determining the mitochondrial function at various stages of heart failure [24,163]. On the basis of clinical observations and general characteristics such as the amount of abdominal fluid accumulation, lung and liver congestion, as well as the heart-to-body weight ratio, different age groups of cardiomyopathic animals were considered at prefailure, early failure, moderate failure, and severe stages of heart failure [24,163]. It can be seen from the data in Table 1 that there was a progressive depression in the high-energy phosphates (both creatine phosphate and ATP) content at early, moderate, and severe stages of heart failure without any significant change at the prefailure stage. However, when the OXPHOS activity, by using pyruvate-malate as a substrate, was examined in the mitochondria isolated from the hearts of cardiomyopathic hamsters at different stages of heart failure, the phosphorylation rate was depressed (without any changes in ADP/O ratio) only at the severe stages of heart failure (Figure 1). The depressed OXPHOS activity and respiratory rate at state 3 in mitochondrial preparations or whole-heart homogenates were also seen by using glutamate- pyruvate or glutamate alone as substrates at the severe stages of heart failure [163] in cardiomyopathic hamsters. Mitochondrial Ca^2+^ uptake activity, unlike mitochondrial ATPase activity, was also found to be decreased at the severe stage of heart failure in cardiomyopathic hamsters (Figure 2). Although these observations indicate a generalized defect in the mitochondrial function at severe stages of heart failure, the observed depression in the high-energy phosphate stores at early and moderate stages of heart failure cannot be explained on the basis of such changes in energy production [163]. It should be mentioned that the energy utilization systems due to myofibrillar ATPase and membrane ATPases at different stages of heart failure were either unaltered or depressed in this experimental model [24,163].

**Figure 3:**
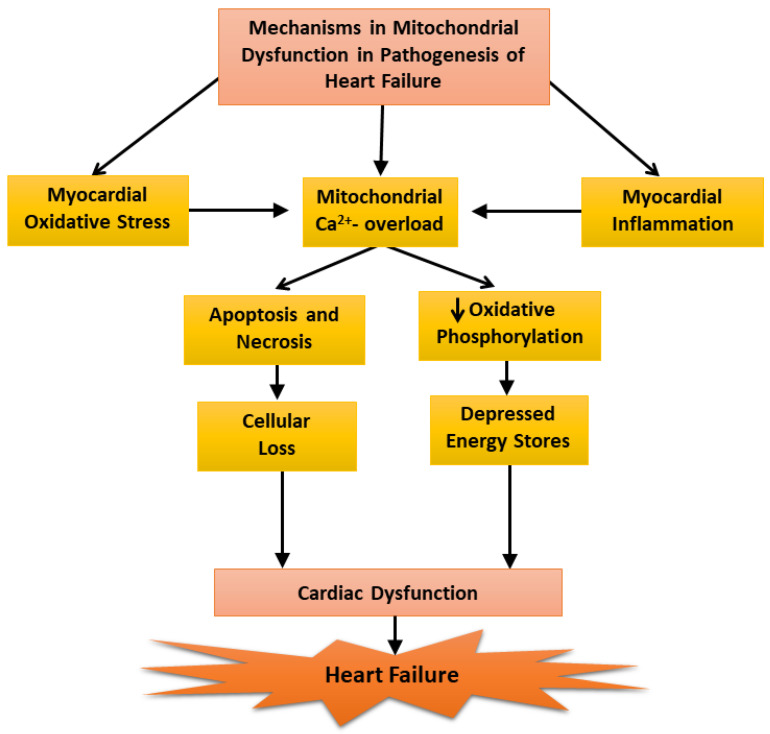
Mechanisms of mitochondrial dysfunction in the pathogenesis of heart failure.

Our inability to demonstrate depression mitochondrial OXPHOS activity at early and moderate stages of heart failure was found to be due to a loss of Ca^2+^ from the mitochondria during the process of isolation because the medium employed for this procedure contained ethylenediamine-tetra acetic acid (EDTA). It should also be mentioned that the mitochondrial Ca^2+^ content (About 10 umol/mg protein), in preparations obtained from the failing hearts were not different from that of the controls [24]. However, when the mitochondria were prepared using an isolation medium in the absence of EDTA, the mitochondrial Ca^2+^ content in the failing hearts were 5 to 7 times higher than that in the control and mitochondrial OXPHOS activity was significantly depressed at the early, moderate, and severe stages of heart failure [24]. These results can be seen to explain the variable defects in mitochondrial OXPHOS activity in heart failure as reported by several investigators. Furthermore, these observations also support the role of mitochondrial Ca^2+^-overload in depressing OXPHOS activity in these organelles [23]. Since ROS are formed due to defects in mitochondrial electron transport in disease hearts [117,145], it is likely that depressed OXPHOS activity observed in cardiomyopathic hamsters at severe stages of heart failure may also be partially due to changes in the electron transport chain complexes. In view of the findings that oxidative stress, myocardial inflammation, and Ca^2+^-handling abnormalities are generally associated with cardiac dysfunction [86,103,104,137], it is proposed that these pathogenic factors may serve as mechanisms for inducing mitochondrial Ca^2+^-overload and the subsequent depression of mitochondrial OXPHOS activity during the development of heart failure (Figure 3). However, it is pointed out that the earliest changes in the mitochondria may be of a compensatory nature for removing cellular stress due to cardiac inflammation and alterations in the substrate metabolism whereas delayed changes including depressed mitochondrial OXPHOS in heart failure may be of an adaptive nature for lowering the ROS production.

## 5. Mitochondrial Targets for Potential Therapeutic Interventions

Since the heart depends heavily on mitochondrial OXPHOS, which accounts for 90% of cellular ATP production, strategies that target defects in this pathway are essential for preserving energy production, enhancing cardiac function, and the survival of heart failure patients. Several therapeutic approaches in this regard involve targeting the mitochondria within the failing heart to modulate the organization of the respiratory complexes into supercomplexes for oxidative phosphorylation [168,169,170,171,172,173,174,175,176]. In coronary artery disease patients, the activities of complexes I, II, and III are depressed in the failing heart, despite an upregulation in protein expression, indicating a functional deficit in OXPHOS-related proteins [177]. Empagliflozin has shown promise as a treatment option to improve cardiac function by increasing OXPHOS, enhancing glucose and fatty acid oxidation as well as promoting cardiac efficiency [178,179,180]. Furthermore, elamipretide associated with cardiolipin has been observed to restore mitochondrial bioenergetics [181] because mitochondrial cardiolipin is essential for the proper assembly and stability of the electron transport chain to ensure the function of OXPHOS [182]. Studies in animal models of chronic heart failure have shown that elamipretide elicited a normalization of mitochondrial function as this agent improved respiration, restored the membrane potential, reduced ROS formation, and enhanced the maximum rate of ATP synthesis. Since the mutation of ribosomal protein S5 (MRPS5/uS5m) in the mitochondria has been observed to result in impaired mitochondrial protein translation and a depressed level of K1f15 protein in the OXPHOS pathway, exogenous Klf15 was found to rescue defects and restore balance to the cardiac metabolome [149]. The impairment of OXPHOS leads to a decline in the function of complexes I, II, and III in individuals with coronary artery disease and those with failing hearts [177,183]. The overexpression of PFK (phosphofructokinase) or the administration of PFKM has been demonstrated to inhibit doxorubicin-induced cardiotoxicity by enhancing glycolysis and the OXPHOS system, and thus PFKM may be considered for developing new treatment for heart failure [184].

There is also experimental evidence to suggest sodium-glucose cotransporter-2 (SGLT2) inhibitors restore the balance between glycolysis and OXPHOS [180], providing significant cardiac protection to patients suffering from heart failure [185]. On the other hand, metformin, which is known to promote glucose uptake and exert beneficial actions in various non-diabetic malignant diseases [186,187], did not show conclusive beneficial effects in non-diabetic patients with coronary heart disease [188]. Nonetheless, it is noteworthy that the mitochondrial antioxidant system can be selectively activated to prevent or treat mitochondrial dysfunction and, in this context, coenzyme Q10, a natural antioxidant, is known to activate the mitochondrial antioxidant system. It is pointed out that a CoQ10 deficiency has been linked to electron transport chain dysfunction and oral supplementation has been reported to reverse this trend [189,190,191,192] as CoQ10 supplementation was observed to improve cardiac function, reduce cardiovascular mortality, and enhance survival rates in heart failure [193]. MitoQ, a compound that mimics Coenzyme Q10, has also shown to be highly effective in protecting against oxidative damage. It is capable of preventing lipid peroxidation and the mitochondrial damage caused by superoxide radicals [194,195]. The therapeutic benefits of MitoQ have been demonstrated in various animals and humans with diabetes, hypertension, and inflammation, in addition to offering protection against oxidative stress and improving the integrity of the cardiac mitochondrial network in heart failure [196,197,198]. Thus, it would be worthwhile to undertake a large double-blind clinical trial to establish the beneficial effects of MitoQ in heart failure. It may also be noted that the inhibition of Drp1 (dynamin-related protein 1) maintains mitochondrial integrity and improves OXPHOS, playing a cardioprotective role during cardiac stress circumstances such as ischemia-reperfusion injury and cardiac arrest in cells by hindering excessive fission at the onset of reperfusion in animal models [199,200,201,202]. Thus, the development of appropriate inhibitors of Drp1 may prove valuable for preserving mitochondrial function in heart failure.

## 6. Conclusions and Perspectives

By virtue of their ability to generate energy as ATP, mitochondria play an important role in maintaining the cardiac structure and function. These organelles produce ATP upon the oxidation of different substrates as well as the OXPHOS system, involving a specialized electron transport chain organized in the form of complexes I to V. While some ATP is transformed into creatine phosphate for the storage of energy in the myocardium, most of ATP is utilized for the contraction–relaxation cycle and maintaining cation homeostasis by myofibrillar ATPase, as well as sarcoplasmic reticulum and sarcolemmal ATPases in cardiomyocytes, respectively. Thus any abnormality in the process of substrate oxidation or any component of the mitochondrial OXPHOS system can be seen to decrease high-energy phosphate stores in the myocardium, resulting in cardiac dysfunction and progression to heart failure. Such a view is not intended to de-emphasize the contribution of other subcellular and molecular defects in the pathogenesis for the development of heart failure.

It is noteworthy that mitochondria have a remarkable ability to accumulate Ca^2+^ and serve as a Ca^2+^-sink to maintain cellular integrity. However, several studies have shown that mitochondrial Ca^2+^-overload is one of the major causative factors for inducing defects in the OXPHOS system under a wide variety of pathological conditions. In fact, defects in the sarcoplasmic and sarcolemmal Ca^2+^-transport systems in the failing hearts have been shown to elicit mitochondrial Ca^2+^-overload. Furthermore, it should be noted that mitochondrial dysfunction is also associated with impaired electron transport for the production of ROS due to changes in any one or all of the complexes in the mitochondrial electron transport chain for the induction of abnormalities in OXPHOS. In addition, the activation of enzymes such as NADPH and monoamine oxidase by vasoactive hormones, which become accumulated in the mitochondria during the development of heart failure, has also been reported to generate ROS. Both mitochondrial Ca^2+^ and ROS not only depress the antioxidant reserve within mitochondria and open mitochondrial pores for the leakage of cytotoxic substances such as cytochrome C, but also provide signals for the development of apoptosis, cellular damage, and subsequent heart failure.

In view of the role of mitochondrial Ca^2+^-overload and mitochondrial ROS generation for depressing the OXPHOS system, it is evident that mitochondrial dysfunction plays a critical role in the depletion of myocardial high-energy phosphate stores during the development of heart failure. Accordingly, several pharmacological and metabolic interventions, which promote the mitochondrial OXPHOS function, have been reported to produce beneficial effects in heart failure. Likewise, different antioxidants, which prevent the generation and effectiveness of mitochondrial ROS, have been shown to delay the progression of heart failure. Thus, there is real challenge to develop specific and safe metabolic and antioxidants interventions (either alone or in combination) targeting the mitochondrial OXPHOS system for the improved therapy of heart failure.

## Figures and Tables

**Table 1:** Creatine phosphate (CP) and adenosine triphosphate (ATP) content in the control and cardiomyopathic hamster (UM- X7.1) hearts at different stages of heart failure.

Age Group	Stage of Heart Failure	CP (μmol Phosphate/g Dry Heart wt)	ATP (μmol Phosphate/g Dry Heart wt)
90 to 280 days	Control	55.4 ± 2.2	22.8 ± 1.8
90 to 100 days	Prefailure	50.3 ± 2.0	21.2 ± 1.9
120 to 160 days	Early Failure	41.1 ± 1.2 *	18.6 ± 2.0
160 to 200 days	Moderate Failure	30.4 ± 1.0 *	16.7 ± 1.5 *
200 to 280 days	Severe Failure	9.7 ± 1.4 *	12.6 ± 1.2 *

Each value for cardiomyopathic hamsters is based on the data in our article [24,163] and is a mean ± SE of four to six experiments, except the values for the corresponding age-matched control batch were grouped together as the values for these age groups were not significantly different (*p* > 0.05) from each other. The heart failure stages in cardiomyopathic hamsters of different age groups were determined on the basis of the formation of ascites, lung wt, and heart/ body ratios as these parameters were increased progressively, whereas liver wt was significantly increased in animals at moderate and severe stages of heart failure [24,163]. * Significantly different (*p* < 0.05) from corresponding control values.
